# Targeting the DNHD1-HDAC6 axis inactivates prostate cancer cell aggressiveness *in vitro*, suggesting potential therapeutic vulnerability

**DOI:** 10.3892/or.2026.9204

**Published:** 2026-09-30

**Authors:** An Ni Lee, Chia Chang Wu, Su Wei Hu, Chih Heng Chen, Yi Te Chiang, Wei Tang Kao, Kai Yi Tzou, Chia Hung Liu, Shao Wei Dong, Yu Ting Wang, Yueh Lin Lee, Siou Bi Li, Che An Wu, Chiao Chun Liao, Ming Hsien Chan, Po Han Chiu, Chien Hsiu Li

**Affiliations:** 1 Department of Urology, Shuang Ho Hospital, Taipei Medical University, New Taipei City, Taiwan 23561, R.O.C.; 2 Department of Urology, School of Medicine, College of Medicine, Taipei Medical University, Taipei, Taiwan 23561, R.O.C.; 3 Taipei Medical University (TMU) Research Center of Urology and Kidney, Taipei Medical University, Taipei City, Taiwan 23561, R.O.C.; 4 Graduate Institute of Biomedical Informatics, College of Medical Science and Technology, Taipei Medical University 110, Taipei, Taiwan 23561, R.O.C.; 5 Department of Tropical Medicine, School of Medicine, College of Medicine, National Yang Ming Chiao Tung University, Taipei, Taiwan 300093, R.O.C.; 6 Department of Biomedical Imaging and Radiological Sciences, National Yang Ming Chiao Tung University, Taipei 112, Taiwan 300093, R.O.C.

**Keywords:** prostate cancer, dynein heavy chain domain 1, histone deacetylase 6, prognosis, bioinformatic

## Abstract

Prostate cancer progression toward an aggressive, therapy-resistant disease state remains a major clinical challenge, indicating a need to identify novel molecular drivers of such progression and actionable therapeutic targets. The present study analyzed the role of dynein heavy chain domain 1 (*DNHD1*), a previously uncharacterized regulator of malignancy, in prostate cancer. Integrative transcriptomic analyses across The Cancer Genome Atlas and independent clinical cohorts revealed that *DNHD1* is markedly upregulated in advanced-stage prostate cancer and positively correlated with increasing Gleason scores. Elevated *DNHD1* expression was associated with poor overall survival and disease-free survival, which supports its clinical relevance as a prognostic biomarker. Functional analyses demonstrated that *DNHD1* promoted tumor cell proliferation, colony formation, migration and invasion *in vitro*. Molecular correlation modeling and pathway analyses revealed histone deacetylase 6 (*HDAC6*) as a key downstream effector of *DNHD1*. *DNHD1* expression was positively correlated with and regulated *HDAC6* expression in clinical datasets. Gain-of-function and loss-of-function experiments further revealed that *HDAC6* is required for *DNHD1*-mediated oncogenic phenotypes in prostate cancer, positioning *HDAC6* as a functional mediator downstream of *DNHD1*. The DNHD1-HDAC6 axis was associated with cilium assembly-related pathways, suggesting a previously unrecognized association between ciliary regulation and prostate cancer progression. HDAC6 inhibition with tubastatin A effectively suppressed *DNHD1*-driven tumor growth and motility, as well as increased acetylated α-tubulin and decreased androgen receptor signaling. These findings indicated a potential therapeutic vulnerability within the DNHD1-HDAC6 axis. They implied that in prostate cancer, the DNHD1-HDAC6 axis, a novel regulatory pathway, promotes aggressiveness and that *HDAC6* is a targetable mediator of *DNHD1*-driven malignancy. This axis can be considered a prognostic indicator and mechanistic foundation for developing targeted therapeutic strategies for advanced prostate cancer.

## Introduction

Prostate cancer is one of the most prevalent malignancies and a leading cause of cancer-related mortality worldwide. In the United States, it is estimated that 333,830 new cases and 36,320 deaths will occur in 2026, with prostate cancer accounting for ~31% of all newly diagnosed cancers in men (1–5). Despite widespread clinical use of prostate-specific antigen (PSA) for prostate cancer diagnosis, its limited specificity and sensitivity frequently result in both false-positive and false-negative results (6–8). Conventional diagnostic approaches, such as digital rectal examination and transrectal ultrasound-guided biopsy, are invasive and insufficient for accurate early detection in clinically ambiguous cases (3). Although advances in surgery and robotics have improved local disease management, mechanistically informed biomarkers and therapeutic targets must be identified to enable accurate detection of prostate cancer progression at the molecular level (9,10). Together, these limitations represent a major gap in the understanding of the regulatory circuits underlying prostate cancer progression and therapeutic resistance. Emerging evidence suggests that prostate cancer progression is governed not only by canonical oncogenic signaling pathways but also by cytoskeletal architecture, intracellular transport systems and primary cilium dynamics. These structural and spatial regulatory systems have increasingly been recognized as higher-order determinants of cellular plasticity, lineage adaptability and metastatic competence. However, the upstream molecular nodes integrating these processes into coordinated oncogenic programs remain poorly defined, representing a major conceptual gap in prostate cancer biology. Dynein heavy chain domain 1 (*DNHD1*) is classified as a member of the coiled-coil domain-containing family, characterized by multiple coiled-coil regions within its protein structure (11). Exome sequencing profiles have revealed potential associations between truncating mutations in *DNHD1* and intellectual disability (12) and between missense mutations and severe fetal anomaly syndromes (13). Furthermore, the biallelic variants of *DNHD1* are associated with male infertility (14,15). *DNHD1* can undergo epigenetic methylation in lead-exposed uterine and brain tissues, which alters its expression levels (16). The importance of *DNHD1* in the brain may be related to its role in signal transmission, particularly as a component of the cytoplasmic dynein dimer (17). In the context of vascular structural functions, *DNHD1* is associated with congenital heart defects (13,18); in particular, *DNHD1* methylation is correlated with cardiac wall thickness (19). Studies using the zebrafish model have demonstrated involvement of DNHD1 in Kupffer's vesicle organogenesis and ciliogenesis (20). In cancer research, mutations in *DNHD1* have been identified in epithelioid glioblastoma and pancreatic cancer; however, their precise biological functions remain unclear (21,22). Although rotundine treatment can inhibit *DNHD1* in colorectal cancer cells (23), its prognostic value and molecular mechanisms in cancer biology, particularly prostate cancer, remain to be elucidated.

Histone deacetylase 6 (*HDAC6*), a cytoplasmic deacetylase, functions as a central regulator of microtubule dynamics, proteostasis and primary cilium disassembly (24–26). In prostate cancer, HDAC6 regulates α-tubulin acetylation and stabilizes androgen receptor (AR) signaling output, promoting tumor growth, lineage plasticity and resistance to androgen deprivation therapy (ADT) (27–29). Notably, HDAC6 has also emerged as a pharmacologically actionable target, with selective inhibitors demonstrating antitumor activity through disruption of cytoskeletal integrity and oncogenic signaling networks (30,31). Despite these advances, the upstream regulatory mechanisms underlying HDAC6 expression and its integration into broader signaling hierarchies in prostate cancer remain unclear.

The present study hypothesized that DNHD1 functions as an upstream regulatory node integrating cytoskeletal organization with HDAC6-dependent oncogenic signaling in prostate cancer. Through integrated analyses of clinical cohorts, transcriptomic datasets and functional assays, the present study identified *DNHD1* as a previously underrecognized promoter of prostate cancer cell aggressiveness. The present study demonstrated that *DNHD1* expression is consistently upregulated in advanced prostate cancer and associated with poor clinical outcomes across independent cohorts. Mechanistically, the present study defined HDAC6 as a downstream effector of *DNHD1* and established a DNHD1-HDAC6 axis that regulates tumor cell proliferation, invasion and metastatic potential. Notably, pharmacological inhibition of HDAC6 effectively suppresses *DNHD1*-mediated oncogenic phenotypes *in vitro*, revealing a potential therapeutic vulnerability within this axis. In addition to providing functional validation, the findings of the present study position DNHD1-HDAC6 signaling as a higher-order regulatory axis integrating cytoskeletal remodeling, ciliary dynamics and AR signaling in prostate cancer. In general, these findings indicated that DNHD1-HDAC6, a previously underrecognized regulatory axis, links structural cellular organization to oncogenic signaling hierarchies in prostate cancer. In particular, the present study suggested that DNHD1 may be associated with prostate cancer cell aggressive phenotypes, providing a preliminary conceptual framework for further exploration of the cytoskeleton-cilium-AR regulatory network in advanced prostate cancer, which warrants future *in vivo* and prospective clinical validation.

## Materials and methods

### Study design and overall analytical framework

The present study integrated transcriptomic analyses, computational network modeling, *in vitro* functional assays and pharmacological perturbation experiments to characterize the DNHD1-HDAC6 axis in prostate cancer. Publicly available datasets were systematically analyzed to evaluate gene expression patterns, clinical associations, functional pathways and prognostic significance. Experimental validation was performed in prostate cancer cell lines with distinct AR and lineage states. A systems biology framework was employed to identify downstream effectors and infer regulatory hierarchies and mechanistic validation and therapeutic targeting were subsequently employed.

### Transcriptomic datasets and cohort selection

Gene expression profiles were obtained from The Cancer Genome Atlas (TCGA)-prostate adenocarcinoma (PRAD) cohort, Gene Expression Omnibus (GEO) (https://www.cancer.gov/ccg/research/genome-sequencing/tcga) and additional independent datasets (GSE21032, GSE35988, GSE48403 and GSE36133: https://www.ncbi.nlm.nih.gov/geo/query/acc.cgi?acc=GSE21032; https://www.ncbi.nlm.nih.gov/geo/query/acc.cgi?acc=GSE35988; https://www.ncbi.nlm.nih.gov/geo/query/acc.cgi?acc=GSE48403; http://www.ncbi.nlm.nih.gov/geo/query/acc.cgi?acc=GSE36133). Cross-platform pancancer datasets, including Cell 2015 (https://www.cbioportal.org/study/summary?id=prad_tcga_pub), Firehose Legacy (https://www.cbioportal.org/study/summary?id=prad_tcga) and PanCancer Atlas (https://www.cbioportal.org/study/summary?id=prad_tcga_pan_can_atlas_2018), were used for comparative oncogenomic analyses. Raw or processed expression matrixes were retrieved and harmonized across datasets by using platform-specific normalization strategies. Log2-transformed expression values were used for downstream analyses when available. Samples were stratified according to clinical annotations, including tumor stage, Gleason score, metastatic status and treatment history.

### Differential expression and pancancer analysis

The differential gene expression between tumor and normal tissues, as well as between early- and late-stage tumors, was assessed using nonparametric statistical frameworks suitable for RNA-sequencing and microarray data distributions. Genes associated with tumor progression were identified through stage-stratified comparisons. Pancancer expression profiling was conducted across multiple epithelial malignancies to determine the tumor-type specificity of *DNHD1* and *HDAC6* dysregulation.

### Clinicopathological association analysis

The present study evaluated associations between gene expression and clinicopathological variables, including pathological T and N stage, Gleason score, residual tumor status and treatment exposure, by using Spearman rank correlation analysis. Stratified subgroup analyses were performed to assess expression trends across disease severity gradients. A two-sided P<0.05 was considered to indicate significance.

### Survival and prognostic modeling

Overall survival (OS) analyses were performed using the online tool Gene Expression Profiling Interactive Analysis (GEPIA2) (http://gepia2.cancer-pku.cn). For the analyses of disease-free interval (DFI) and progression-free interval (PFI) in the TCGA-PRAD cohort, as well as shorter disease-free survival (DFS) in the GSE21032 dataset, raw clinical data were extracted from the respective supplementary tables and analyzed using Kaplan-Meier survival models. Patients were stratified into high- and low-expression groups on the basis of median gene expression thresholds, unless otherwise specified. Hazard ratios (HR) and 95% confidence intervals (Cis) were estimated using Cox proportional hazards regression models. Combined gene signature analyses were performed using dual-gene stratification models to assess additive prognostic effects.

### Correlation-based systems biology and network construction

To construct *DNHD1*-associated regulatory networks, correlation analyses were performed across the TCGA-PRAD and multiple independent prostate cancer transcriptomic cohorts. Genes with Spearman's ρ≥0.3 or Spearman's ρ≤-0.3 relative to *DNHD1* were defined as *DNHD1*-associated gene sets. To increase robustness and reduce cohort-specific bias, the present study generated integrated gene lists by using intersection analysis across multiple independent datasets. The resulting gene networks were subjected to downstream pathway enrichment analysis.

### Pathway enrichment and functional annotation

Functional enrichment analysis was performed using ingenuity pathway analysis (IPA). Canonical pathways, upstream regulatory networks and disease and function annotations were evaluated on the basis of enrichment significance scores (−log_10_ P). Biological processes associated with cytoskeletal remodeling, vesicular trafficking, organelle organization, metabolic reprogramming and cilium assembly were specifically interrogated. In Gene Ontology classification, the present study focused on structural cellular organization pathways, particularly those related to microtubule dynamics and ciliary regulation.

### Construction of DNHD1-HDAC6 coregulatory gene signature

Genes positively correlated with both DNHD1 and HDAC6 were identified through Venn-based integration across transcriptomic datasets. To ensure inclusion of only protein-coding genes, pseudogenes and long noncoding RNAs were filtered out. The resulting gene set, defined as the DNHD1-HDAC6 downstream transcriptional signature, was used for correlation analysis and survival modeling.

### Cell lines and culture conditions

Human prostate cancer cell lines representing different AR and lineage states were used: LNCaP (cat. no. BCRC 60088), PC-3 (cat. no. BCRC 60122), 22RV1 (cat. no. BCRC 60545) and DU145 (cat. no. BCRC 60348) cells lines were purchased from the Bioresource Collection and Research Center. Cells were maintained under standard conditions (37°C; 5% CO_2_) in appropriate culture media supplemented with fetal bovine serum and antibiotics (32,33). Cell line identity was consistent with American Type Culture Collection or validated repositories and cells were routinely tested for contamination.

### Generation of stable knockdown and overexpression models

*DNHD1, E2F6* and *HDAC6* expression was modulated using a 2nd-generation lentiviral shRNA-mediated knockdown and cDNA overexpression systems (Table SI). Lentiviral particles were generated by co-transfecting HEK-293T cells (obtained from the Bioresource Collection and Research Center; cat. no. BCRC 60019) with pLKO.1-shRNA or pLenti6/V5-DEST-cDNA transfer plasmids, alongside the packaging plasmid pCMVΔR8.9 and envelope plasmid pMD.G at a mass ratio of 4:3:1 (total 10 µg plasmid per 10-cm dish: 5 µg transfer plasmid, 3.75 µg pCMVΔR8.9, and 1.25 µg pMD.G) using Lipofectamine^®^ 3000 (Thermo Fisher Scientific, Inc.).

Lentiviral supernatants were harvested at 48 and 72 h post-transfection, filtered through a 0.45-µm membrane filter, and stored at −80°C. To establish stable cell populations, target cells were transduced at a multiplicity of infection (MOI) of 5 in the presence of 8 µg/ml polybrene. Stable knockdown cell lines were selected with 2 µg/ml puromycin for 3 days and maintained in 0.5 µg/ml puromycin. Stable overexpression cell lines were selected with 5 µg/ml blasticidin for 4 days and maintained in 2 µg/ml blasticidin. Subsequent experiments were performed 48 h post-selection. Knockdown and overexpression efficiency were validated using reverse transcription-quantitative (RT-q) PCR and immunoblotting (32,33).

### RNA extraction, RT-qPCR and chromatin immunoprecipitation

Target cells were seeded at a density of 1×10^6^ cells/well prior to RNA extraction. Total RNA extraction, cDNA synthesis, and qPCR assays were performed strictly according to the manufacturers' protocols. Total RNA was isolated and reverse transcription was performed to generate cDNA using the TOOLSQuant II Fast RT Kit (cat. no. KRT-BA06-2; BIOTOOLS Co., Ltd.). Gene expression levels were quantified using SYBR Green-based qPCR assays with PowerTrack SYBR Green Master Mix (cat. no. A46012; Applied Biosystems; Thermo Fisher Scientific, Inc.; Table SII). Relative expression was calculated using the 2^−ΔΔCq^ method (34), with housekeeping genes as internal normalization controls. For promoter binding analysis, potential *E2F6*-binding motifs within the *HDAC6* promoter region were identified using a bioinformatic sequence prediction tool (JASPAR database). A putative E2F6-binding site (5′-GAGAGGGAAGA-3′, positions −781 to −770 bp relative to the transcription start site) was identified within the HDAC6 promoter. Chromatin immunoprecipitation was performed using the EZ-Magna ChIP A/G kit (MilliporeSigma) according to the manufacturer's instructions. In brief, target cells were harvested at a density of 1×107 cells per ChIP reaction and cross-linked with 1% formaldehyde (MilliporeSigma) to fix protein-DNA complexes. Chromatin was sheared by sonication using an Ultrasonic Disruptor UD-201 (TOMY Digital Biology Co., Ltd.; 15 cycles of 30 sec on/30 sec off at 4°C) to yield DNA fragments ranging from 200–1,000 bp, which were verified by agarose gel electrophoresis. A portion of the clarified lysate (10%) was saved as Input DNA for normalization. Cell lysates (100 µg per reaction) were immunoprecipitated with anti-E2F6 (2 µg/reaction; cat. no. 31464-1-AP; Proteintech Group, Inc.) or control IgG (2 µg/reaction; cat. no. 30000-0-AP; Proteintech Group, Inc.) overnight (12–16 h) at 4°C with magnetic A/G beads. Immunocomplexes were washed sequentially with Low Salt, High Salt, LiCl and TE Wash Buffers provided in the kit. Specific promoter DNA fragments encompassing the predicted *E2F6* binding motif (positions −781 to −770 bp) were subsequently amplified and quantified through qPCR using PowerTrack SYBR Green Master Mix (cat. no. A46012; Applied Biosystems; Thermo Fisher Scientific, Inc.). The thermocycling conditions were: Initial denaturation at 95°C for 3 min; 35 cycles of denaturation at 95°C for 30 sec, annealing at 58°C for 30 sec and elongation at 72°C for 30 sec; followed by a final extension at 72°C for 5 min. The ChIP enrichment efficiency was determined using the Percent Input approach. Briefly, the Cq value of the 10% Input fraction was adjusted for dilution [ΔCq (Input)=Cq (Input)-log2(10)], and the percent input was calculated using the formula: % Input=100×2[^ΔCq (Input)-Cq [ChIP)]^. Signal acquisition and quantitative processing were carried out using QuantStudio Design and Analysis Software (version 1.3; Applied Biosystems; Thermo Fisher Scientific, Inc.).

### Protein extraction and immunoblotting

Whole-cell lysates were prepared using TOOLS RIPA Lysis Buffer (cat. no. TAAR-ZBZ5; BIOTOOLS Co., Ltd.) supplemented with protease and phosphatase inhibitors. Protein concentrations were determined using the Bio-Rad Protein Assay Kit II (cat. no. 5000002; Bio-Rad Laboratories, Inc.) based on the Bradford method.

Total protein (30–60 µg per lane) was separated through sodium dodecyl sulfate-polyacrylamide gel electrophoresis on 12% polyacrylamide gels and transferred onto polyvinylidene difluoride membranes. These membranes were blocked with 5% non-fat milk in Tris-buffered saline with 0.1% Tween-20 for 1 h at room temperature. Membranes were then incubated overnight at 4°C with antibodies against DNHD1 (cat. no. PAU491Hu01; CLOUD-CLONE CORP), E2F6 (cat. no. 31464-1-AP; Proteintech Group, Inc.), HDAC6 (cat. no. 67250-1-lg; Proteintech Group, Inc.), α-tubulin (cat. no. 2144; Cell Signaling Technology, Inc.), acetylated α-tubulin (cat. no. 3971; Cell Signaling Technology, Inc.), AR (cat. no. 54653; Cell Signaling Technology, Inc.) and PSA (cat. no. 2475; Cell Signaling Technology, Inc.). Followed primary antibody incubation, membranes were washed and incubated with horseradish peroxidase (HRP)-conjugated secondary antibodies, including Goat Anti-Rabbit IgG (H+L) HRP (1:5,000; cat. no. GTX213110-01; GeneTex, Inc.) or Goat Anti-Mouse IgG HRP (1:5,000; cat. no. GTX213111-01; GeneTex, Inc.), for 1 h at room temperature. Protein signals were detected using chemiluminescence and quantified, as necessary. Protein bands were visualized using SuperKine West Femto Maximum Sensitivity Substrate (cat. no. BMU102-EN; Abbkine Scientific Co., Ltd.; distributed by Proteintech Group, Inc.) and captured using the Bio-Rad ChemiDoc MP Imaging System (Bio-Rad Laboratories, Inc.). Protein bands were visualized using SuperKine West Femto Maximum Sensitivity Substrate (cat. no. BMU102-EN.; distributed by Proteintech Group, Inc.) and captured using the Bio-Rad ChemiDoc MP Imaging System (Bio-Rad Laboratories, Inc.). Densitometric analysis was performed using Image Lab Software (version 6.1; Bio-Rad Laboratories, Inc.), with β-Actin (cat. no. GTX26276; GeneTex, Inc.) serving as the internal loading control for normalization.

### Cell proliferation and clonogenic assays

For cell proliferation assays, target cells were seeded into 96-well plates at a density of 2×10^3^ cells/well and cultured at 37°C in a humidified incubator containing 5% CO_2_. Cell proliferation was evaluated using AlamarBlue Cell Viability Reagent (cat. no. DAL1025; Invitrogen; Thermo Fisher Scientific, Inc.) strictly following the manufacturer's protocol. Briefly, AlamarBlue reagent was added to each well (10% of total volume), and cells were incubated for 2–4 h at 37°C. Fluorescence/absorbance was measured using a microplate reader at defined time points. For colony formation assays, cells were seeded into 6-well plates at a density of 2×10^3^ cells/well and cultured at 37°C in a 5% CO_2_ atmosphere for 14 days to allow colony development. Following incubation, culture media were discarded, and cells were fixed with a methanol-acetic acid mixture (7:1, v/v) for 30 min at room temperature. The fixed cells were subsequently stained with 0.1% crystal violet solution (MilliporeSigma) for 30 min at room temperature and gently rinsed with distilled water. Stained plates were imaged, and colonies were captured under a stereomicroscope. Colony quantification was performed using ImageJ software (version 1.53t; National Institutes of Health) via the Analyze Particles plugin after thresholding.

### Migration and invasion assays

Cell migration and invasive capacities were evaluated using a 48-well microchemotaxis Boyden chamber system (Neuro Probe, Inc.) to assess cell motility and invasive capacity. For the invasion assays, polycarbonate membranes with an 8-µm pore size (Neuro Probe, Inc.) were pre-coated with Matrigel (BD Biosciences; Corning, Inc.; diluted 1:8 in serum-free medium) at 37°C for 1 h to form a reconstituted basement membrane. Uncoated membranes were used for migration assays. Target cells were harvested, washed, and resuspended in serum-free culture medium. A total of 2×104 cells in 50 µl of serum-free medium (with or without specified drug treatments) were loaded into the upper chamber wells. The lower chamber wells were filled with 25–30 µl of culture medium supplemented with 10% fetal bovine serum (FBS; Thermo Fisher Scientific, Inc.) as a chemoattractant. The chamber was assembled and incubated at 37°C in a humidified 5% CO_2_ atmosphere for 24 h (migration) or 48 h (invasion). After incubation, non-migrated cells on the upper surface of the membrane were gently scraped off using a cotton swab. Cells that migrated or invaded cells were fixed with 4% paraformaldehyde (or methanol-acetic acid 7:1) for 15–30 min at room temperature and stained with Giemsa stain solution (cat. no. G5637; MilliporeSigma) for 20–30 min at room temperature. Stained membranes were mounted, and migratory/invasive cells were visualized and captured using an inverted light microscope (Olympus Corporation). Cell numbers were quantified across three randomly selected high-power microscopic fields per well using ImageJ software (version 1.53t; National Institutes of Health) (32,33).

### Pharmacological inhibition of HDAC6

To selectively inhibit HDAC6 catalytic activity, human prostate cancer LNCaP cells were seeded in multi-well plates and allowed to adhere overnight at 37°C in a humidified atmosphere containing 5% CO_2_. Cells were then treated with Tubastatin A (cat. no. HY-13271A; MedChemExpress) at indicated concentrations (5 and 10 µM) or with dimethyl sulfoxide (DMSO; MilliporeSigma) as the vehicle control for 24 h at 37°C. The final concentration of DMSO in the culture medium was maintained below 0.1% (v/v) across all experimental groups. The efficacy and specificity of enzymatic HDAC6 inhibition were verified by evaluating the hyperacetylation levels of its downstream substrate, acetylated α-tubulin, via immunoblotting.

### Epistasis and rescue experiments

To establish functional dependency, combinatorial gain-of-function and loss-of-function experiments were performed in prostate cancer cells (LNCaP, PC-3) using a lentiviral gene delivery system. For DNHD1 overexpression, prostate cancer cells were transduced with recombinant lentiviruses encoding full-length DNHD1 cDNA cloned into the pLenti6/V5-DEST Gateway vector (cat. no. V49610; Invitrogen; Thermo Fisher Scientific, Inc.) or an empty destination control vector. Transductions were performed at 37°C for 24 h in the presence of 8 µg/ml Polybrene (hexadimethrine bromide; MilliporeSigma). To establish stable DNHD1-overexpressing populations, cells were selected with Blasticidin (5.0 µg/ml; cat. no. A1113903; Gibco; Thermo Fisher Scientific, Inc.) for 5–7 days. Subsequently, DNHD1-overexpressing cells were transduced with lentiviral particles expressing specific short hairpin RNA targeting HDAC6 (sh-HDAC6; pLKO.1-shHDAC6) or a non-targeting scramble control shRNA (sh-NC) at an optimized multiplicity of infection (MOI=10-20) with 8 µg/ml Polybrene at 37°C for 24 h. Transduced cells were co-selected with Puromycin (2.0 µg/ml; MilliporeSigma) for 48–72 h. Overexpression and knockdown efficiencies were validated at 48–72 h post-transduction via RT-qPCR and immunoblotting (using an anti-V5 tag antibody for exogenous DNHD1 detection). Transduced cells were harvested and re-seeded into downstream functional assays to evaluate rescue outcomes, including cell proliferation (48 h readout), clonogenicity (14 days incubation), and Boyden chamber migration/invasion assays (24–48 h incubation) under standard conditions (37°C; 5% CO_2_).

### Statistical analysis

All statistical analyses were conducted using R (version 4.3.1; R Foundation for Statistical Computing, Vienna, Austria). Data were presented as means ± standard deviations, unless otherwise stated. Between-group comparisons were performed using unpaired Student's t-test, depending on relevant distribution assumptions. Correlation was assessed using Spearman rank correlation analysis. Survival significance was assessed using log-rank tests and Cox proportional hazards regression models. P<0.05 was considered to indicate a statistically significant difference.

## Results

### Dysregulated DNHD1 expression is correlated with unfavorable prostate cancer prognosis

To identify genes associated with advanced prostate cancer and to determine progression-associated transcriptional alterations, transcriptomic profiles from TCGA-PRAD) cohort were stratified by tumor stage (T2a vs. T4). Differential expression analysis revealed a gene subset markedly upregulated in higher-stage tumors; among these genes, *DNHD1* was consistently enriched (Fig. 1A), suggesting that it is involved in prostate cancer progression rather than in tumor maintenance alone. In the TCGA-PRAD cohort, *DNHD1* expression was markedly elevated in tumor tissues compared with adjacent nontumor tissues (P=0.0003; Fig. 1B; Table SIII), further corroborating its association with malignant transformation. Pancancer analysis demonstrated that *DNHD1* upregulation occurs not only in prostate cancer but also across various epithelial malignancies, including cholangiocarcinoma, colon adenocarcinoma (COAD), head and neck squamous cell carcinoma, renal clear cell carcinoma (KIRC), hepatocellular carcinoma and rectal adenocarcinoma (Fig. 1C). However, prognostic stratification analysis revealed that *DNHD1*-associated high-risk clinical outcomes were most consistently observed in PRAD, KIRC and COAD (Fig. 1D), suggesting that *DNHD1* has a context-dependent functional relevance in tumor progression rather than a universal oncogenic role. Notably, *DNHD1* expression was significantly and positively correlated with Gleason score in prostate cancer tissues (P<0.0001; Fig. 1E). Moreover, it increased progressively across stratified Gleason grading groups (Fig. 1F; Table SIV), suggesting that *DNHD1* expression is strongly associated with histopathological severity and tumor aggressiveness. To validate these findings across independent datasets, multiple external prostate cancer cohorts (GSE21032, GSE35988 and GSE48403) were analyzed (Fig. 1G; Table SV). Consistent with the observations in TCGA-PRAD, *DNHD1* expression was markedly upregulated in GSE21032. Elevated *DNHD1* expression was also observed in both localized prostate cancer and metastatic castration-resistant prostate cancer (mCRPC) tissues, suggesting its persistence throughout disease progression and metastatic evolution. Notably, ADT was associated with further upregulation of *DNHD1* expression, indicating its potential involvement in therapy-adaptive transcriptional reprogramming. Survival analysis using TCGA-PRAD cohort data demonstrated that higher *DNHD1* expression was significantly associated with poorer overall survival (OS; P=0.039, HR=3.8) and shorter disease-free survival (DFS; P=3.7×10^−5^; HR=2.4; Fig. 1H). These prognostic associations were independently validated in the GSE21032 cohort, where *DNHD1* expression also correlated with shorter DFS (P=0.0034, HR=3.443, 95% CI=1.494–7.931; Fig. 1I; Table SVI). Taken together, these results suggest that in prostate cancer, *DNHD1* is not only differentially expressed but also associated with a transcriptional program linked to progression, histopathological severity and adverse clinical outcomes. These consistent associations across independent cohorts further corroborate the potential role of *DNHD1* as a progression-associated molecular indicator in prostate cancer.

### DNHD1 promotes prostate cancer cell growth and metastatic potential through tumor cell plasticity regulation

The oncogenic role of *DNHD1* in prostate cancer remains largely undefined. To investigate its potential functional relevance, the present study first examined *DNHD1* expression across prostate cancer cell lines by using the CCLE dataset GSE36133 (Table SVII). Notably, *DNHD1* expression demonstrated a lineage-associated distribution, with lower expression in AR-positive luminal-like cell lines (LNCaP and 22RV1) but higher expression in AR-negative, more aggressive and mesenchymal-like cell lines (PC-3 and DU145; Fig. 2A). Thus, *DNHD1* may be associated with lineage plasticity and a more dedifferentiated tumor state in prostate cancer. Protein-level validation through immunoblotting across prostate cancer cell lines further confirmed this differential expression pattern (Fig. 2B), corroborating the transcriptional and translational consistency of *DNHD1* deregulation in distinct prostate cancer cellular contexts. To investigate its functional role, the present study generated stable *DNHD1*-knockdown and -overexpression models by using lentiviral short hairpin RNA (shRNA) and cDNA systems. On the basis of the endogenous expression profiles, LNCaP and PC-3 cells were used for gain-of-function and loss-of-function experiments, respectively. Efficient modulation of DNHD1 expression was validated by RT-qPCR and immunoblotting (Fig. 2C and D).

Functional assay results demonstrated a potent regulatory role of *DNHD1* in prostate cancer cell function. *DNHD1* knockdown markedly impaired proliferative capacity in PC-3 cells, whereas ectopic *DNHD1* expression considerably enhanced proliferation in LNCaP cells, as indicated by the Alamar Blue assay results (Fig. 2E). These findings were further corroborated by colony formation assay results: *DNHD1* knockdown suppressed long-term clonogenic survival, whereas *DNHD1* overexpression promoted robust colony expansion (Fig. 2F), indicating a critical role for DNHD1 in sustaining the proliferative and survival capacities of prostate cancer cells. Considering the clinical relevance of metastatic dissemination in prostate cancer progression, the present study subsequently evaluated the regulatory role of *DNHD1* in prostate cancer cell motility and invasive behavior. Fibronectin- and Matrigel-based Boyden chamber assays revealed that *DNHD1* knockdown markedly reduced both migratory and invasive potential in PC-3 cells; by contrast, *DNHD1* overexpression led to considerable improvements in LNCaP cell motility and invasion (Fig. 2G and H). These findings indicate that *DNHD1* functionally enhances both proliferative and metastatic phenotypes in prostate cancer cells, consequently establishing DNHD1 as a functional regulator of prostate cancer cell growth and motility. Notably, the associations observed between *DNHD1* expression and AR status indicate a potential link between *DNHD1* and prostate cancer lineage plasticity: In prostate cancer cells, *DNHD1* upregulation may promote a shift toward more aggressive, AR-independent states with enhanced metastatic competence.

### HDAC6 is a key downstream effector within the DNHD1-associated oncogenic network in prostate cancer

To elucidate the downstream regulatory network associated with DNHD1 in prostate cancer, the present study performed a correlation-based system-level analysis using transcriptomic datasets from TCGA-PRAD, Cell 2015, Firehose Legacy and PanCancer Atlas cohorts (35–38). Genes with a robust positive correlation (Spearman's ρ≥0.3) or negative correlation (Spearman's ρ≤-0.3) with *DNHD1* expression were integrated across datasets to construct a high-confidence DNHD1-associated gene signature (Fig. 3A; Table SVIII). Functional enrichment analysis of 2,145 positively correlated and 463 negatively correlated genes conducted using IPA revealed that *DNHD1*-associated transcriptional programs are strongly enriched in pathways regulating cytoskeletal organization, vesicular trafficking, organelle biogenesis and cellular metabolic rewiring (Fig. 3B; Table SIX). Notably, among the top-ranked canonical pathways, cilium assembly emerged as a consistently enriched biological process, highlighting a potential association between *DNHD1* and structural signaling regulation in prostate cancer. Within this cilium-associated regulatory network, *HDAC6* was identified as a biologically and clinically relevant candidate downstream effector. HDAC6 is a well-established cytoplasmic deacetylase, regulating microtubule dynamics through α-tubulin deacetylation. It plays a key role in primary cilium disassembly, protein homeostasis and oncogenic stress adaptation. Notably, although HDAC6 is implicated in prostate cancer progression and AR signaling regulation (39), its position within upstream regulatory networks remains unclear. In the TCGA-PRAD cohort, HDAC6 expression was positively correlated with *DNHD1* expression (R=0.4; P=3.1×10^−20^; Fig. 3C). The result of the network-based analysis therefore suggested that *HDAC6* represents a functional convergence node, linking *DNHD1*-associated transcriptional programs to cytoskeletal and ciliary remodeling pathways in prostate cancer. To validate the clinical relevance of this association, the present study subsequently examined *HDAC6* expression in TCGA-PRAD and independent prostate cancer cohorts. *HDAC6* expression was significantly upregulated in primary tumor tissues compared with in the adjacent nontumor tissues (P=0.0230; Fig. 3D; Table SX). Notably, *HDAC6* expression was strongly and positively correlated with *DNHD1* expression across multiple independent datasets, including TCGA-PRAD (Spearman's ρ=0.4283; P=0.0092) and validation cohorts (Fig. 3E; Table SX), confirming the robustness of this regulatory association. In clinical specimens, higher *HDAC6* expression was positively correlated with a higher Gleason score, suggesting a role in prostate cancer progression and aggressiveness (Fig. 3F; Table SIV). Moreover, comparative analysis across these specimens revealed a significant positive correlation between DNHD1 and HDAC6 expression (Spearman's ρ=0.3436; P<0.0001; Fig. 3G; Table SIV), confirming a coordinated transcriptional relationship between the two molecules. Consistently, elevated HDAC6 expression was observed across localized prostate cancer (GSE21032) and mCRPC (GSE35988) cohorts, further indicating its association with advanced prostate cancer. Notably, ADT led to further upregulation of *HDAC6* expression (GSE48403), suggesting that *HDAC6* is involved in the progression to ADT-adaptive tumor states (Fig. 3H; Table SV). Survival analysis revealed that high *HDAC6* expression was significantly associated with poor OS (P=0.047; HR=4.4) and DFS (P=0.039; HR=1.5) in the TCGA-PRAD cohort (Fig. 3I). The relationship between *HDAC6* expression and DFS was independently validated in the external cohort GSE21032 (P=0.0453; HR=2.332; 95% CI=1.025–5.306). Notably, combined stratification based on *DNHD1* and *HDAC6* expression revealed a high-risk patient subgroup with the poorest clinical outcomes across all cohorts (P<0.0001), suggesting additive or synergistic prognostic value for both genes (Fig. 3J; Table SVI). Together, these results indicate that HDAC6 may be a key downstream effector within the *DNHD1*-associated transcriptional network, affording a model in which DNHD1-HDAC6 signaling constitutes a coordinated regulatory axis governing cytoskeletal remodeling, ciliary dynamics and prostate cancer progression. In prostate cancer, this axis may not only be correlated with tumor aggressiveness and therapy adaptation but also define a clinically relevant high-risk molecular subclass of the condition.

### DNHD1 promotes malignancy in prostate cancer through HDAC6-dependent regulatory mechanisms

To further delineate the mechanistic relationship between DNHD1 and HDAC6 in prostate cancer, the present study first evaluated *HDAC6* expression across prostate cancer cell lines by using CCLE transcriptomic datasets (GSE36133). It was observed that *HDAC6* expression exhibited a lineage-associated distribution, with lower expression in AR-positive luminal-like prostate cancer cells than in AR-negative prostate cancer cell lines (Fig. 4A; Table SVII). This distribution closely parallels *DNHD1* expression patterns, further suggesting a potential functional coupling between DNHD1 and HDAC6 within various prostate cancer cell states. Additionally, integrative correlation analysis revealed a strong positive association between *DNHD1* and *HDAC6* expression across prostate cancer datasets (Spearman's ρ=0.90; Fig. 4B; Table SVII), corroborating a strong transcriptional association between the two genes. To determine whether *DNHD1* functionally regulates *HDAC6* expression, the present study performed gain-of-function and loss-of-function experiments in prostate cancer cell models. qPCR and immunoblotting analyses revealed that *DNHD1* overexpression markedly upregulated *HDAC6* mRNA and protein expression in LNCaP cells, whereas *DNHD1* knockdown considerably downregulated *HDAC6* mRNA and protein expression in PC-3 cells (Fig. 4C and D). These findings suggested that *DNHD1* positively regulates *HDAC6* expression at both the transcriptional and the translational level. Notably, reciprocal perturbation experiments revealed that modulating *HDAC6* expression did not affect *DNHD1* expression (Fig. 4E), indicating a unidirectional regulatory hierarchy in which *DNHD1* acts upstream of *HDAC6*. These results establish *HDAC6* as a downstream effector rather than a feedback regulator within the *DNHD1* signaling axis. To assess the functional contribution of *HDAC6* in prostate cancer progression, the present study established *HDAC6* gain-of-function and loss-of-function models in prostate cancer cell lines (Fig. 4F and G). Consistent with a previous report (29), *HDAC6* knockdown markedly suppressed proliferation and clonogenic growth in PC-3 cells, whereas *HDAC6* overexpression enhanced proliferative capacity in LNCaP cells (Fig. 4H and I). Moreover, Boyden chamber assays revealed that in both cell lines, *HDAC6* knockdown considerably reduced migratory and invasive potential, whereas ectopic *HDAC6* expression promoted aggressive motility (Fig. 4J and K). These results confirmed that *HDAC6* expression is essential and sufficient to drive prostate cancer cell growth and metastatic behavior. To establish whether *HDAC6* functionally mediates *DNHD1*-driven oncogenic phenotypes, the present study performed combinatorial epistasis experiments. *DNHD1* overexpression substantially enhanced proliferation, colony formation, migration and invasion in LNCaP cells. Notably, simultaneous silencing of *HDAC6* and *DNHD1* markedly attenuated all *DNHD1*-induced oncogenic phenotypes (Fig. 4L-O), indicating that *HDAC6* is required for the complete execution of *DNHD1*-mediated tumor-promoting effects.

Upstream regulator analysis of *DNHD1*-simulated networks revealed E2F6 as a potential transcription factor involved in the transactivation of *HDAC6* under *DNHD1* regulation (P=0.0398; Fig. S1A). Transcriptomic profiling of TCGA-PRAD cohort data revealed that *E2F6* expression was positively correlated with the expression of both *DNHD1* (R=0.22; P=1.2×10^−6^) and *HDAC6* (R=0.29; P=1.0×10^−10^). Clinical outcome analysis further revealed that in patients with prostate cancer, *E2F6* upregulation led to significantly worse OS than low *E2F6* expression did (P=0.046; HR=3.8; Fig. S1B). To investigate whether E2F6 functionally modulates *HDAC6* expression, the present study performed gain-of-function and loss-of-function experiments. *E2F6* overexpression considerably upregulated *HDAC6* expression in LNCaP cells, whereas *E2F6* knockdown substantially downregulated *HDAC6* mRNA and protein expression, as validated through qPCR and immunoblotting (Fig. S1C and D). To evaluate whether E2F6 directly transactivates *HDAC6*, the present study performed promoter sequence analysis and identified a putative E2F6-binding motif (5′-GAGAGGGAAGA-3′) located between positions −781 and −770 bp within the *HDAC6* promoter (Fig. S1E). Chromatin immunoprecipitation coupled with qPCR demonstrated an enriched recruitment of E2F6 to the *HDAC6* promoter relative to the immunoglobulin G (IgG) control; this effect was further enhanced after ectopic *E2F6* expression in LNCaP cells (Fig. S1F). Thus, E2F6 drives *HDAC6* transactivation by directly binding to the *HDAC6* promoter. Given the regulatory hierarchy established in the aforementioned text, the present study further explored the functional role of E2F6 in the DNHD1-HDAC6 axis. Immunoblotting revealed that *DNHD1* overexpression stimulated E2F6 expression, whereas *E2F6* knockdown suppressed *DNHD1* expression (Fig. S1G), suggesting a reciprocal regulatory relationship between DNHD1 and E2F6. Functional assays revealed that *E2F6* knockdown markedly attenuated *DNHD1*-induced proliferation, migration and invasion in LNCaP cells (Fig. S1H-J). By contrast, although E2F6 transactivated *HDAC6, HDAC6* knockdown slightly downregulated *E2F6* expression (Fig. S1K). The results of epistatic rescue experiments demonstrated that *HDAC6* knockdown substantially reversed the enhancements in colony-forming, migratory and invasive capacities conferred by *E2F6* overexpression (Fig. S1L-N), indicating that *HDAC6* is an indispensable downstream mediator of E2F6-driven malignant phenotypes.

These results further confirmed the presence of a DNHD1-HDAC6 axis in prostate cancer cells, in which *DNHD1* is an upstream regulator that activates *HDAC6* through E2F6 to promote proliferation and motility in tumor cells *in vitro*. Notably, *HDAC6* is a critical epistatic mediator of *DNHD1*-driven malignancy, indicating that the DNHD1-HDAC6 axis is a functionally coherent regulatory module determining prostate cancer aggressiveness.

### Pharmacological targeting of the DNHD1-HDAC6 axis reveals a therapeutically actionable vulnerability in prostate cancer

Given the vital role of *HDAC6* in regulating microtubule dynamics, proteostasis and oncogenic signaling networks, its selective inhibition has emerged as a promising cancer treatment strategy (24–26). To evaluate the translational relevance of the DNHD1-HDAC6 axis in prostate cancer, the present study employed tubastatin A, a well-characterized selective HDAC6 inhibitor. In LNCaP cells, tubastatin A treatment resulted in a dose-dependent increase in acetylated α-tubulin levels (Fig. 5A), confirming that the enzymatic activity of HDAC6 was effectively inhibited. Time-course analysis further revealed sustained accumulation of acetylated α-tubulin after tubastatin A exposure, indicating strong suppression of HDAC6-dependent deacetylation activity (Fig. 5B). Notably, HDAC6 inhibition was accompanied by suppression of downstream AR signaling output, as indicated by decreased AR and PSA levels (Fig. 5C). Thus, the enzymatic activity of HDAC6 may functionally contribute to maintenance of AR-driven transcriptional programs in prostate cancer cells. Functionally, tubastatin A markedly impaired prostate cancer cell viability, clonogenic capacity, migratory behavior and invasive potential, demonstrating that pharmacological inhibition of HDAC6 effectively abrogates key malignant phenotypes associated with cancer progression (Fig. 5D-G). These results establish HDAC6 as a critical regulator of prostate cancer growth and metastatic competence. Notably, pharmacological inhibition of HDAC6 also attenuated *DNHD1*-driven oncogenic phenotypes. In *DNHD1*-overexpressing LNCaP cells, tubastatin A treatment effectively reversed *DNHD1*-induced upregulation of HDAC6 expression and suppressed associated AR and PSA expression (Fig. 5H). These findings indicate that *DNHD1*-mediated signaling is functionally dependent on the enzymatic activity of HDAC6. Combinatorial functional assays further revealed that HDAC6 inhibition considerably abrogated *DNHD1*-induced enhancement of proliferation, colony formation, migration and invasion (Fig. 5I-L). This epistatic evidence confirms that HDAC6 is required for the execution of *DNHD1*-driven cancer-promoting programs.

These results indicated that the DNHD1-HDAC6 axis is a pharmacologically tractable signaling module in prostate cancer. In addition to holding prognostic relevance, this pathway represents a functional therapeutic vulnerability, whereby HDAC6 inhibition can efficiently suppress *DNHD1*-driven tumor growth and metastatic behavior. Thus, targeting the DNHD1-HDAC6 axis is a potential therapeutic strategy for advanced prostate cancer.

### Association of the DNHD1-HDAC6 axis with prostate cancer pathophysiology and clinical progression

To further delineate the clinical relevance of the DNHD1-HDAC6 axis, the present study elucidated its association with established pathological parameters and disease progression metrics in the TCGA-PRAD cohort. At the gene level, *DNHD1* and *HDAC6* expression demonstrated a consistent positive correlation across multiple pathological strata, including the N stage (Spearman's ρ=0.3308; P<0.0001; Fig. 6A) and T stage (Spearman's ρ=0.3203; P<0.0001; Fig. 6B), suggesting coordinated upregulation during prostate cancer progression. Notably, this coregulatory pattern persisted in residual tumor specimens, where *DNHD1* expression remained positively correlated with *HDAC6* expression (Spearman's ρ=0.3290; P<0.0001; Fig. 6C), indicating that the DNHD1-HDAC6 axis is maintained even in posttreatment disease contexts. Clinically, both *DNHD1* and *HDAC6* expression individually stratified patients with significantly worse disease-free interval (DFI) and progression-free interval (PFI). Higher *DNHD1* expression was associated with a considerably poorer DFI (HR=5.733; P<0.0001); moreover, higher *HDAC6* expression was associated with a considerably worse PFI (HR=2.157; P=0.0309; Fig. 6D and E; Table SXI). Notably, integrated analysis of the combined DNHD1-HDAC6 signature further enhanced prognostic discrimination, identifying a subgroup of patients with the poorest DFI (P<0.0001; Fig. 6F; Table SXI). A similar trend was observed for PFI: High-risk patients with markedly decreased survival probabilities demonstrated high expression of both *DNHD1* and *HDAC6* (Fig. 6G-I; Table SXII). Together, these findings indicated that the DNHD1-HDAC6 axis provides more additive prognostic information than single-gene models do, supporting its role as a coordinated molecular module rather than a set of independent biomarkers.

To further characterize the transcriptional architecture associated with the DNHD1-HDAC6 axis, the present study identified a set of 625 positively correlated genes through Venn-based integration of *DNHD1*- and *HDAC6*-associated expression profiles (Fig. 6J; Table SXIII). Pathway enrichment analysis of this gene set revealed that despite partial divergence from single-gene pathway outputs, cilium assembly remained the most consistently enriched biological process. Thus, in prostate cancer, ciliary regulation may represent a convergent functional output of DNHD1-HDAC6 signaling. After excluding non-protein-coding transcripts, the present study defined a refined 570-gene signature as the downstream transcriptional program of the DNHD1-HDAC6 axis (Fig. 6K; Table SXIV). This signature was strongly and positively correlated with both *DNHD1* (Spearman's ρ=0.75; P<7.5×10^−89^) and *HDAC6* (Spearman's ρ=0.58, P<1.2×10^−45^; Fig. 6L; Table SXV), indicating robust transcriptional coupling across independent patient samples. Notably, this axis-associated gene program also demonstrated significant prognostic value, stratifying patients with regard to OS (P=0.028; HR=4.3) and DFS (P=0.0062; HR=1.8; Fig. 6M).

To further assess whether the DNHD1-HDAC6 axis is an independent prognostic indicator in prostate cancer, the present study performed a multivariate Cox proportional hazards regression analysis including *DNHD1, HDAC6* and *KLK3* as covariates across multiple clinical endpoints (Table I). In the GSE21032 validation cohort (n=113), the independent risk factors for shortened DFS were identified to be *DNHD1* (HR=1.015; 95% CI=1.001–1.028; P=0.0334) and *HDAC6* (HR=1.012; 95% CI=1.003–1.020; P=0.0073). Consistent with these findings, evaluation within the larger TCGA-PRAD cohort revealed that the overall Cox models for both DFI and PFI were highly significant. In particular, shortened DFI was predicted by elevated expression of *DNHD1* (HR=1.804; 95% CI=1.233–2.640; P=0.0024) and *HDAC6* (HR=3.883; 95% CI=1.059–14.233; P=0.0407), independent of *KLK3* expression. Moreover, both genes demonstrated strong independent prognostic value for PFI, with a 1.54-fold and 2.24-fold increase in progression risk conferred by *DNHD1* (HR=1.541; 95% CI=1.238–1.919; P=0.0001) and *HDAC6* (HR=2.238; 95% CI=1.139–4.395; P=0.0194), respectively. These multivariate analysis results confirmed that the DNHD1-HDAC6 axis affords a robust, independent molecular prognostic framework for predicting disease recurrence and progression in prostate cancer.

Taken together, these results support a systems-level model in which *DNHD1* and *HDAC6* operate as a coordinated regulatory axis that establishes a stable transcriptional state associated with tumor progression, ciliary dysregulation and adverse clinical outcomes in prostate cancer. Rather than acting as isolated biomarkers, *DNHD1* and *HDAC6* appear to converge on a shared downstream gene network that denotes a biologically and clinically coherent program underlying prostate cancer aggressiveness.

## Discussion

The present study identified *DNHD1* as a regulator of malignancy in prostate cancer and defined the DNHD1-HDAC6 axis that functionally promotes tumor cell aggressiveness *in vitro*. In contrast to studies focused on individual prognostic biomarkers, the present study integrated clinical, molecular and functional evidence to position *DNHD1* not only as a correlative marker of prostate cancer but also as a potential contributor to its aggressiveness. Notably, the present study also observed that the DNHD1-HDAC6 axis is regulatory and pharmacologically targetable at the cellular level, thereby providing meaningful mechanistic insight and revealing the potential translational relevance of our findings.

*DNHD1* has not been previously characterized in the context of prostate cancer. Integrative analyses revealed that *DNHD1* expression is consistently elevated in advanced disease stages, is positively correlated with Gleason score progression and predicts unfavorable clinical outcomes across multiple independent cohorts. Functional analyses further demonstrated that *DNHD1* directly promotes tumor cell proliferation, clonogenicity, migration and invasion *in vitro*. These results were consistent with a model in which *DNHD1* may contribute to malignancy in prostate cancer cells rather than functioning as a passive biomarker alone.

Mechanistically, *HDAC6* was noted to be a critical downstream effector of *DNHD1*. *HDAC6* has been extensively implicated in cytoskeletal remodeling, protein quality control and ciliary disassembly; all these processes have increasingly been recognized as integral to tumor progression (40,41). In prostate cancer, *HDAC6* can regulate microtubule dynamics through deacetylation of α-tubulin and can modulate AR stability and transcriptional activity, thereby contributing to tumor progression and therapy resistance (27,42–44). However, the upstream regulatory mechanisms underlying *HDAC6* expression in prostate cancer remain unclear. The current findings indicated that *DNHD1* regulates *HDAC6* expression at both transcriptional and protein levels and that *HDAC6* is required for *DNHD1*-mediated oncogenic phenotypes. Reciprocal rescue experiments further established a functional hierarchy in which *HDAC6* acts downstream of *DNHD1*, reinforcing its role as a key mediator of *DNHD1*-driven malignancy.

Pathway analysis further revealed a prominent enrichment of cilium assembly programs associated with the DNHD1-HDAC6 axis. Given the emerging role of primary cilia as critical signaling hubs for pathways such as the Hedgehog and Wnt pathways, ciliary homeostasis disruption has increasingly been associated with cancer progression (45–49). In addition, studies have suggested that ciliary dynamics may influence AR signaling and cellular adaptation to androgen-deprived conditions (50–52). The current findings suggested that *DNHD1*-driven activation of *HDAC6* may promote tumor aggressiveness, at least in part, by modulating ciliary dynamics. They thereby reveal a previously underexplored association between cilium-associated processes and prostate cancer pathobiology. In general, these observations indicated that the DNHD1-HDAC6 axis contributes to coordinated regulation of cytoskeletal remodeling, ciliary function and oncogenic signaling.

The association between the DNHD1-HDAC6 axis and AR signaling is another notable observation of the current study. The present study observed that *DNHD1* and *HDAC6* expression levels are upregulated under androgen-deprived conditions and in castration-resistant prostate cancer, suggesting they play roles in adaptive responses to prostate cancer treatment. Consistently, pharmacological inhibition of *HDAC6* attenuated AR and PSA expression, indicating that the DNHD1-HDAC6 axis may interfere with AR signaling networks. These findings suggested that DNHD1-HDAC6 signaling contributes to therapeutic resistance, particularly in the context of ADT and is a mechanism underlying prostate cancer progression toward castration-resistant states.

Notably, the current results indicated a therapeutically exploitable vulnerability within the DNHD1-HDAC6 axis. Pharmacological targeting of *HDAC6* has emerged as a promising strategy in cancer treatment; moreover, certain *HDAC6* inhibitors can disrupt cytoskeletal dynamics, impair protein homeostasis and modulate oncogenic signaling pathways. In prostate cancer, *HDAC6* inhibition can interfere with AR signaling and reduce cancer cell viability, indicating its potential clinical relevance (27,28,53,54). In this context, the present study provided additional mechanistic and functional support for targeting *HDAC6*: Tubastatin A effectively suppressed *DNHD1*-driven tumor growth and motility and attenuated AR signaling output. As DNHD1 was identified as an upstream regulator of HDAC6 *in vitro*, the present study hypothesized that DNHD1 expression might represent a potential candidate marker to explore in future studies regarding responsiveness to HDAC6-targeted strategies, though this hypothesis requires independent *in vivo* and prospective clinical validation. This may provide a preliminary framework for biomarker-informed patient selection. Thus, the DNHD1-HDAC6 axis may be leveraged not only as a therapeutic target but also as a framework for precision medicine in prostate cancer.

This study has several critical limitations. First, although the *in vitro* functional assays provided strong mechanistic findings, they were not validated in animal models (such as xenograft tumor growth or metastasis models). As prostate cancer progression, invasion and therapeutic responses are highly cross-gated by the complex *in vivo* tumor microenvironment, the current findings, from a simplified model, should be interpreted with caution in terms of their implications regarding systemic progression. In particular, direct clinical comparisons of *DNHD1* and *HDAC6* expression levels in tissue samples from benign prostate, localized prostate cancer, high-Gleason-score prostate cancer, mCRPC and post-ADT prostate cancer tissues remain to be experimentally established. Thus, the current study should be considered to provide preliminary evidence positioning the DNHD1-HDAC6 axis merely as a candidate biomarker requiring independent clinical validation, rather than a clinically applicable biomarker at this stage. Second, the precise molecular mechanism through which *DNHD1* regulates *HDAC6* expression (such as whether this regulation occurs through direct transcriptional control or indirect signaling pathways) warrants further elucidation. Third, although pathway analysis implicated cilium-associated processes associated with the DNHD1-HDAC6 axis, direct functional validation of ciliary dynamics is warranted to establish their causal contribution to prostate cancer progression. Future studies should integrate rigorous *in vivo* animal models and independent, multistage prospective clinical cohorts to validate the therapeutic potential and clinical applicability of targeting the DNHD1-HDAC6 axis in advanced prostate cancer.

In summary, the present study identified a novel DNHD1-HDAC6 axis associated with aggressive prostate cancer cell phenotypes *in vitro*, though its potential effect on clinical outcomes warrants further *in vivo* and prospective clinical validation. These findings broaden the current understanding of prostate cancer biology by linking *DNHD1*, a novel upstream regulator, to *HDAC6*-dependent oncogenic pathways and cilium-associated processes. Moreover, the *in vitro* findings suggested that this regulatory axis may represent a potential candidate target, providing a preliminary conceptual foundation that requires further *in vivo* and prospective clinical validation to evaluate its therapeutic potential in advanced prostate cancer.

## Supplementary Material

Supporting Data

Supporting Data

## Figures and Tables

**Figure 1.**
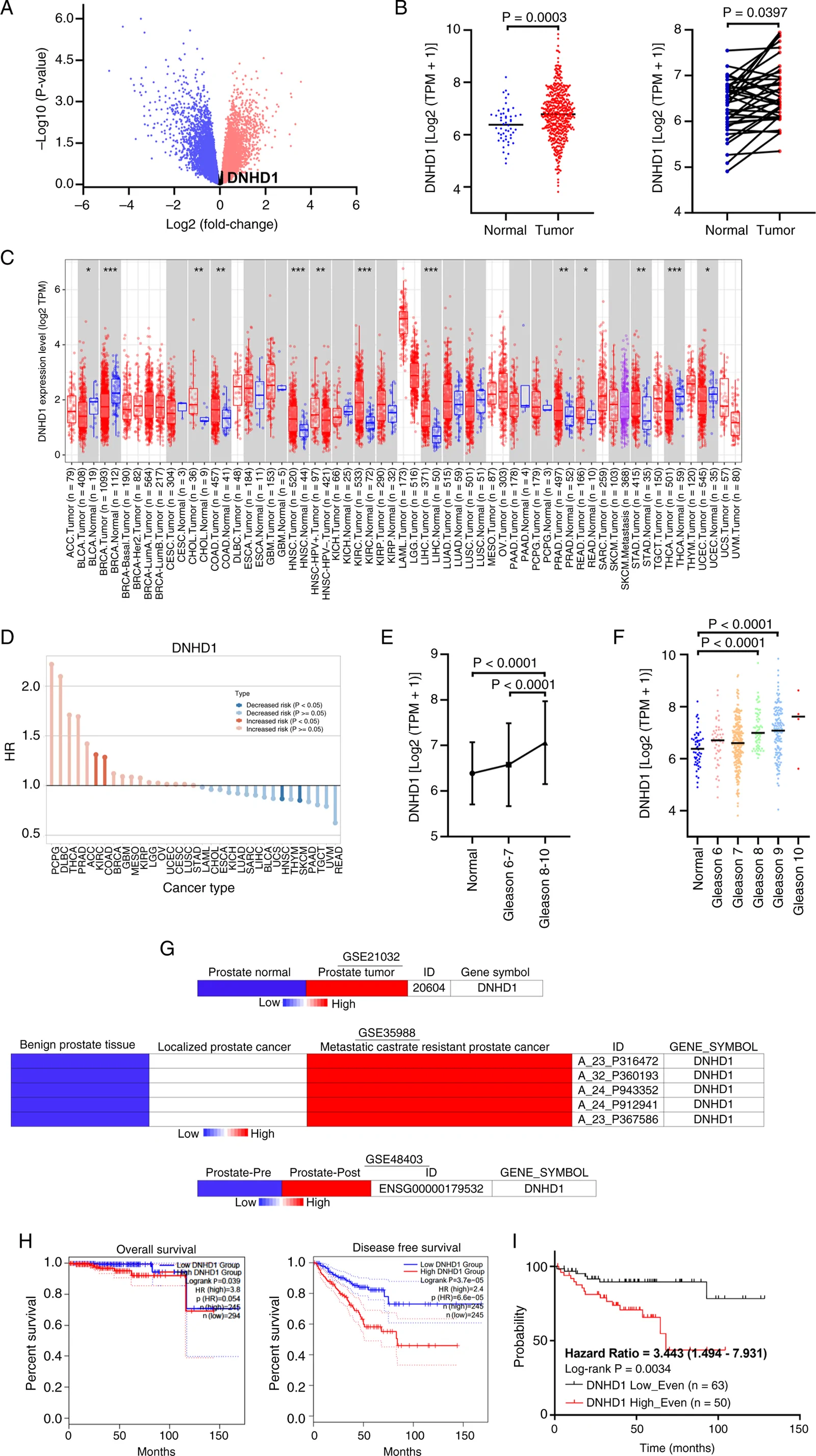
Dysregulation of *DNHD1* expression is associated with prostate cancer progression and unfavorable clinical outcomes. (A) Differential expression analysis of TCGA-PRAD transcriptomic profiles stratified by pathological stage (T2a vs. T4), indicating *DNHD1* as one of the transcripts markedly enriched in advanced prostate cancer. (B) *DNHD1* expression levels in paired prostate tumor and adjacent nontumor tissues from the TCGA-PRAD cohort, indicating increased *DNHD1* expression in tumor specimens. (C) Pancancer expression profiling of *DNHD1* across TCGA-PRAD cohorts, indicating differential expression patterns among multiple human cancer types. (D) Pancancer prognostic analyses of association between *DNHD1* expression and clinical outcome across distinct tumor types, indicating that PRAD, KIRC and COAD are tumor entities with significant *DNHD1*-associated prognostic stratification. (E) Αnalysis between *DNHD1* expression and Gleason score in prostate cancer specimens. (F) Distribution of *DNHD1* expression across stratified Gleason grade score in the TCGA-PRAD cohort. (G) Validation of *DNHD1* expression patterns in independent prostate cancer cohorts (GSE21032, GSE35988 and GSE48403), including benign prostate tissues as well as localized prostate cancer, mCRPC and ADT-associated prostate cancer tissues. (H) Kaplan-Meier survival analyses of OS and DFS according to *DNHD1* expression status in the TCGA-PRAD cohort. (I) Independent validation of prognostic significance of *DNHD1* expression for DFS in GSE21032. *P<0.05, **P<0.01, ***P<0.001. DNHD1, dynein heavy chain domain 1; TCGA, The Cancer Genome Atlas; TPM, transcripts per million; mCRPC, metastatic castration-resistant prostate cancer; ADT, androgen deprivation therapy; OS, overall survival; DFS, disease-free survival; HR, hazard ratio; GEO, Gene Expression Omnibus; ACC, Adrenocortical Carcinoma; BLCA, Bladder Urothelial Carcinoma; BRCA, Breast Invasive Carcinoma; CESC, Cervical Squamous Cell Carcinoma and Endocervical Adenocarcinoma; CHOL, Cholangiocarcinoma; COAD, Colon Adenocarcinoma; DLBC, Lymphoid Neoplasm Diffuse Large B-cell Lymphoma; ESCA, Esophageal Carcinoma; GBM, Glioblastoma Multiforme; HNSC, Head and Neck Squamous Cell Carcinoma; KICH, Kidney Chromophobe; KIRC, Kidney Renal Clear Cell Carcinoma; KIRP, Kidney Renal Papillary Cell Carcinoma; LAML, Acute Myeloid Leukemia; LGG, Brain Lower Grade Glioma; LIHC, Liver Hepatocellular Carcinoma; LUAD, Lung Adenocarcinoma; LUSC, Lung Squamous Cell Carcinoma; MESO, Mesothelioma; OV, Ovarian Serous Cystadenocarcinoma; PAAD, Pancreatic Adenocarcinoma; PCPG, Pheochromocytoma and Paraganglioma; PRAD, Prostate Adenocarcinoma; READ, Rectum Adenocarcinoma; SARC, Sarcoma; SKCM, Skin Cutaneous Melanoma; STAD, Stomach Adenocarcinoma; TGCT, Testicular Germ Cell Tumors; THCA, Thyroid Carcinoma; THYM, Thymoma; UCEC, Uterine Corpus Endometrial Carcinoma; UCS, Uterine Carcinosarcoma; UVM, Uveal Melanoma.

**Figure 2.**
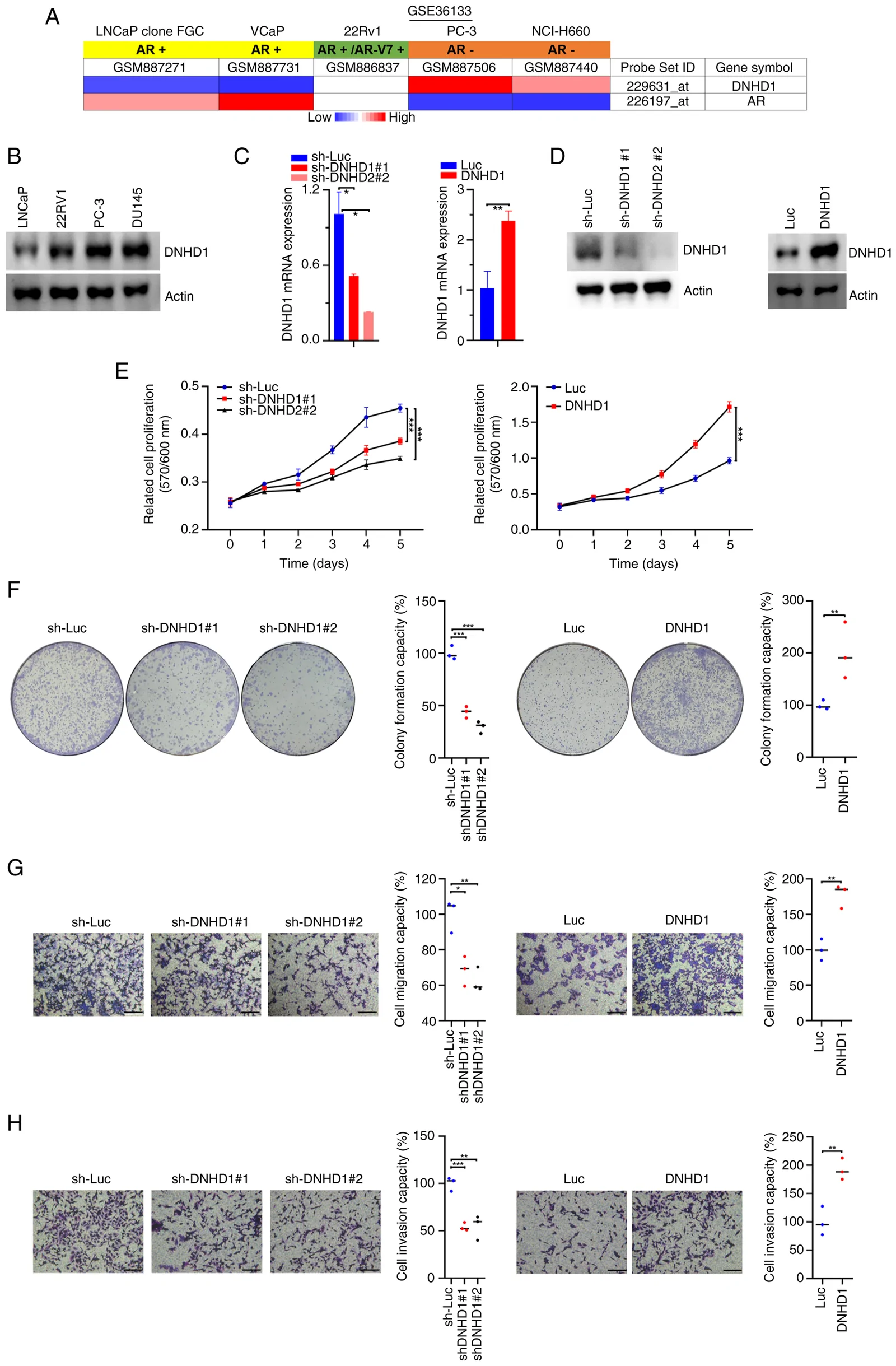
*DNHD1* facilitates prostate cancer cell growth and metastatic behavior. (A) Analysis of *DNHD1* expression across prostate cancer cell lines from the CCLE dataset (GSE36133), including AR-positive (LNCaP and 22RV1) and AR-negative (PC-3 and DU145) cell models. (B) Immunoblotting of DNHD1 expression in a panel of prostate cancer cell lines. (C and D) Establishment and validation of *DNHD1* gain-of-function and loss-of-function models. *DNHD1* overexpression in LNCaP cells and *DNHD1* knockdown in PC-3 cells were confirmed through qPCR and immunoblotting. (E) Alamar Blue proliferation assays indicating effects of *DNHD1* overexpression in LNCaP cells and *DNHD1* knockdown in PC-3 cells on cell growth. (F) Colony formation assays assessing clonogenic capacity after *DNHD1* overexpression in LNCaP cells or *DNHD1* knockdown in PC-3 cells. (G) Boyden chamber migration assays for migratory activity in *DNHD1*-overexpressing LNCaP cells and *DNHD1*-knockdown PC-3 cells. (H) Matrigel invasion assays for invasive capacity of *DNHD1*-overexpressing LNCaP cells and DNHD1-knockdown PC-3 cells. (Scale bar, 100 µm). *P<0.05, **P<0.01, ***P<0.001. DNHD1, dynein heavy chain domain 1; CCLE, Cancer Cell Line Encyclopedia; AR, androgen receptor; qPCR, quantitative polymerase chain reaction; shRNA, short hairpin RNA; sh-Luc, short hairpin RNA targeting luciferase (control); Luc, luciferase; AR-V7, androgen receptor splice variant 7; GSE, Gene Expression Omnibus Series.

**Figure 3.**
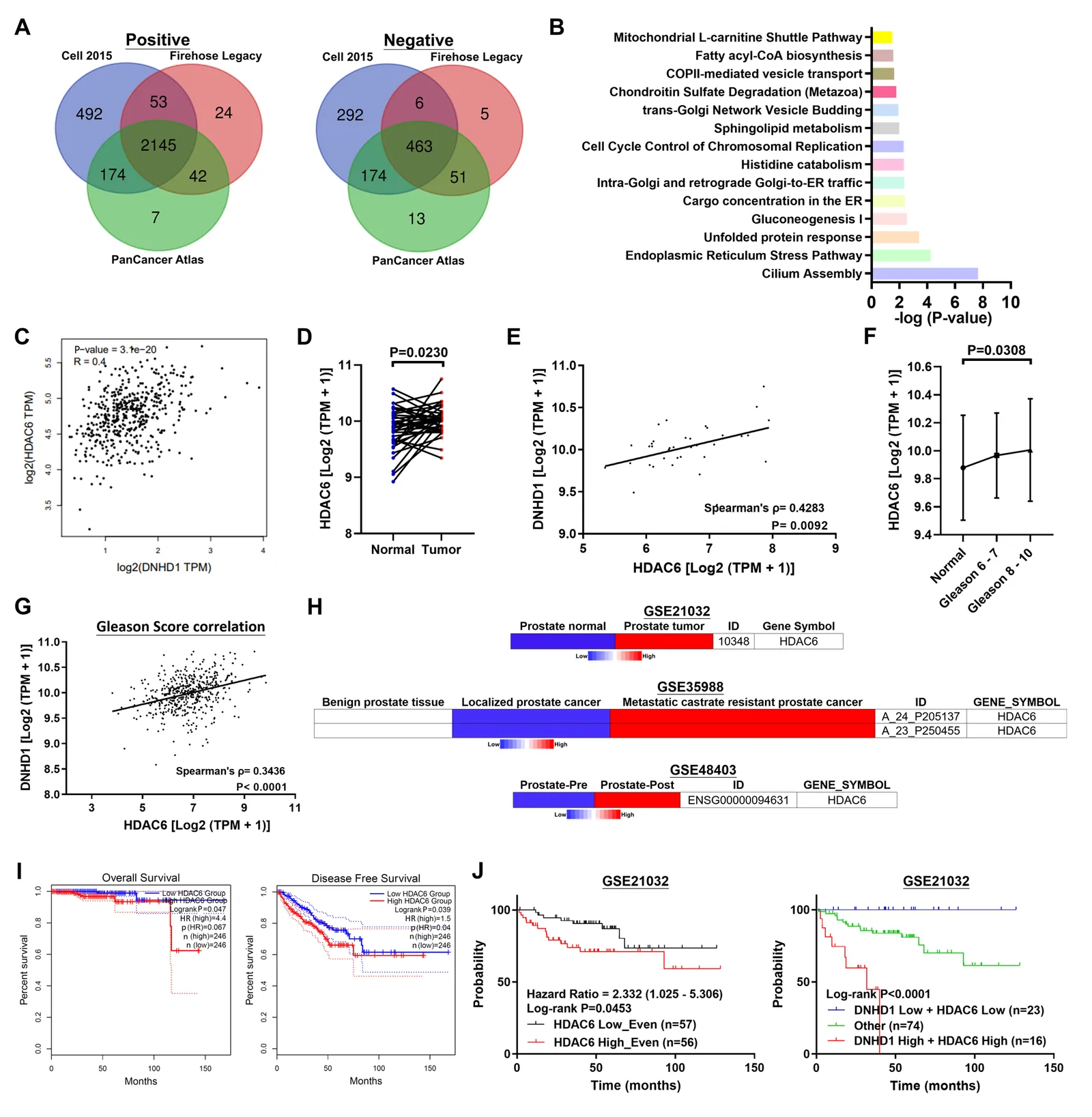
*HDAC6* is a key downstream effector within the *DNHD1*-associated oncogenic network in prostate cancer. (A) Integrative correlation analysis across the TCGA-PRAD, Cell 2015, Firehose Legacy and PanCancer Atlas cohorts to identify genes consistently associated with *DNHD1* expression (Spearman's ρ≥0.3 or Spearman's ρ≤-0.3), generating a high-confidence *DNHD1*-associated transcriptional network. (B) IPA of *DNHD1*-associated genes revealing significant enrichment of biological processes related to cytoskeletal organization, vesicular transport, organelle dynamics and metabolic regulation. Cilium assembly appeared to be one of the most consistently enriched pathways, implicating *DNHD1* in structural signaling programs associated with prostate cancer progression. (C and D) Transcriptomic analyses of TCGA-PRAD specimens demonstrating increased *HDAC6* expression in prostate cancer tissues relative to normal prostate tissues. Representative comparisons and quantitative analyses are presented. (E) Correlation analysis in the TCGA-PRAD cohort. The significant positive association between *DNHD1* and *HDAC6* expression confirmed a coordinated transcriptional relationship between these genes in clinical prostate cancer specimens. (F) Clinicopathological analysis revealing a positive association between *HDAC6* expression and Gleason score, thereby indicating progressive upregulation of *HDAC6* expression with increasing histological aggressiveness. (G) Independent validation within prostate cancer patient cohorts, confirming a significant positive correlation between *DNHD1* and *HDAC6* expression levels, supporting the robustness of the DNHD1-HDAC6 axis. (H) Validation across independent clinical datasets, demonstrating elevated *HDAC6* expression in localized prostate cancer, mCRPC and ADT-treated prostate cancer tissues, indicating its association with disease progression and therapy-adaptive tumor states. (I) Kaplan-Meier survival analyses demonstrating that upregulated *HDAC6* expression is associated with inferior OS and DFS in patients with prostate cancer, supporting its prognostic relevance. (J) Combined stratification based on *DNHD1* and *HDAC6* expression. A high-risk molecular subgroup with the poorest clinical outcome was noted, confirming the additive prognostic value of the DNHD1-HDAC6 axis. HDAC6, histone deacetylase 6; DNHD1, dynein heavy chain domain 1; TCGA, The Cancer Genome Atlas; PRAD, prostate adenocarcinoma; IPA, Ingenuity Pathway Analysis; COPII, coat protein complex II; ER, endoplasmic reticulum; TPM, transcripts per million; mCRPC, metastatic castration-resistant prostate cancer; ADT, androgen deprivation therapy; OS, overall survival; DFS, disease-free survival; HR, hazard ratio; GEO, Gene Expression Omnibus; GSE, Gene Expression Omnibus Series.

**Figure 4.**
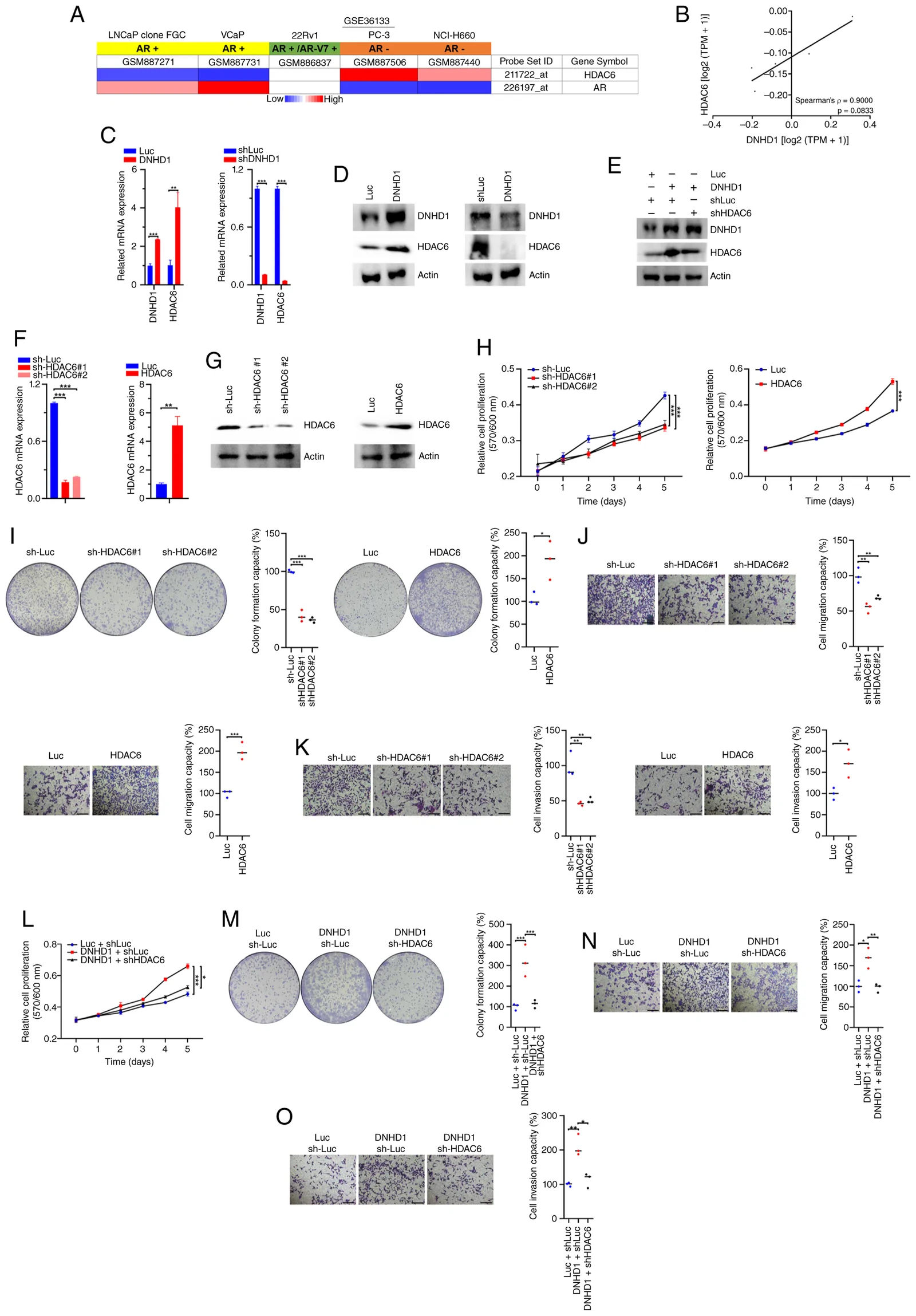
*DNHD1* promotes malignancy in prostate cancer cells through *HDAC6*-dependent regulatory mechanisms. (A) Analysis of the CCLE-derived prostate cancer transcriptomic dataset GSE36133, demonstrating a lineage-associated distribution pattern of *HDAC6* expression, with lower expression observed in AR-positive luminal-like prostate cancer cell lines relative to AR-negative cell lines. (B) Correlation analysis across prostate cancer datasets, revealing a strong positive association between *DNHD1* and *HDAC6* expression (Spearman's ρ=0.90), confirming a coordinated transcriptional relationship between these two molecules. (C and D) Positive regulatory effect of *DNHD1* on *HDAC6* transcript and protein expression. Ectopic expression of *DNHD1* in LNCaP cells increased *HDAC6* expression, whereas *DNHD1* knockdown in PC-3 cells reduced *HDAC6* mRNA and protein expression, as determined through qPCR and immunoblotting. (E) Reciprocal perturbation analyses demonstrating that modulating HDAC6 expression did not alter *DNHD1* expression, indicating a unidirectional regulatory relationship in which *DNHD1* functions as an upstream regulator of *HDAC6*. (F and G) Establishment and validation of *HDAC6* gain-of-function and loss-of-function models in prostate cancer cells. *HDAC6* overexpression in LNCaP cells and *HDAC6* knockdown in PC-3 cells were confirmed through qPCR and immunoblotting. Functional analyses demonstrating that *HDAC6* contributes to prostate cancer cell growth and clonogenic capacity. *HDAC6* overexpression (H) enhanced proliferative and (I) colony-forming ability in LNCaP cells, whereas *HDAC6* knockdown suppressed these phenotypes in PC-3 cells. (J and K) Boyden chamber migration and Matrigel invasion assays demonstrating that *HDAC6* positively regulates prostate cancer cell motility and invasive behavior. Ectopic *HDAC6* expression increased migratory and invasive capacities in LNCaP cells, whereas *HDAC6* knockdown markedly impaired these phenotypes in PC-3 cells. (L-O) Epistasis analyses in *DNHD1*-overexpressing LNCaP cells indicating that *HDAC6* is required for DNHD1-driven oncogenic phenotypes. *DNHD1* promoted cell proliferation, clonogenic growth, migration and invasion, whereas concurrent *HDAC6* silencing markedly attenuated these tumor-promoting effects (Scale bar, 100 µm). *P<0.05, **P<0.01, ***P<0.001. DNHD1, dynein heavy chain domain 1; HDAC6, histone deacetylase 6; CCLE, Cancer Cell Line Encyclopedia; AR, androgen receptor; AR-V7, androgen receptor splice variant 7; TPM, transcripts per million; qPCR, quantitative polymerase chain reaction; mRNA, messenger RNA; shRNA, short hairpin RNA; sh-Luc, short hairpin RNA targeting luciferase (control); Luc, luciferase; shHDAC6, short hairpin RNA targeting histone deacetylase 6; GSE, Gene Expression Omnibus Series.

**Figure 5.**
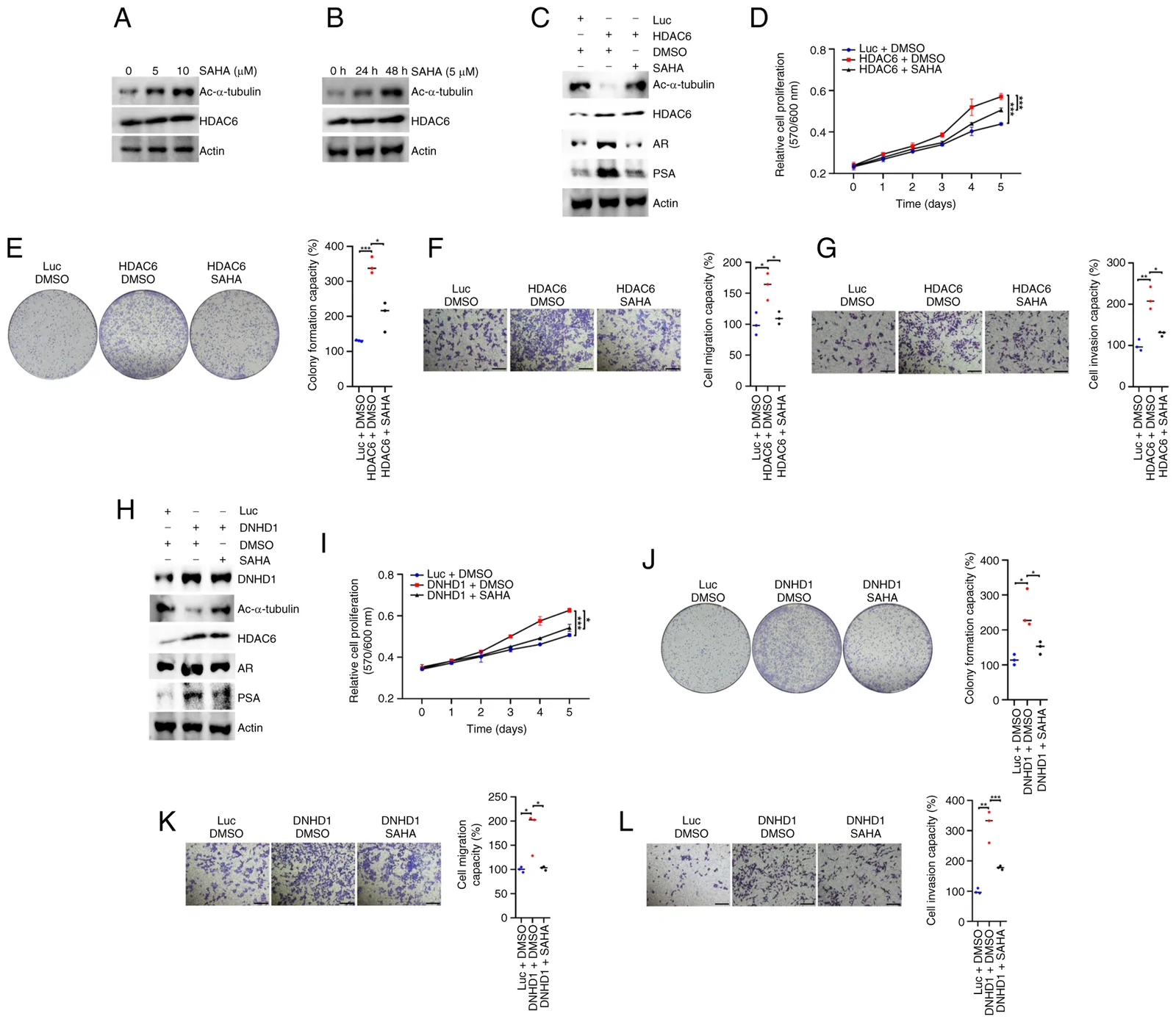
Pharmacological inhibition of *HDAC6* suppresses *DNHD1*-driven oncogenic phenotypes in prostate cancer. (A) Immunoblotting of LNCaP cells treated with increasing concentrations of the selective HDAC6 inhibitor tubastatin A. The results indicate dose-dependent accumulation of acetylated α-tubulin, confirming inhibition of HDAC6 deacetylase activity. (B) Time-course analysis in LNCaP cells demonstrating sustained increase in acetylated α-tubulin levels after tubastatin A treatment, thereby indicating prolonged suppression of *HDAC6*-dependent deacetylation. (C) Pharmacological inhibition of *HDAC6*. The results demonstrate reduced AR and PSA expression in LNCaP cells, consistent with suppression of AR signaling activity. (D-G) Functional analyses in LNCaP cells demonstrating that tubastatin A treatment markedly inhibited cell viability, clonogenic growth, migratory capacity and invasive behavior. (H) Immunoblotting in *DNHD1*-overexpressing LNCaP cells. The results indicate that tubastatin A treatment suppressed *DNHD1*-associated *HDAC6* expression and concomitantly reduced AR and PSA expression. (I-L) Functional epistasis analyses in *DNHD1*-overexpressing LNCaP cells. The results demonstrated that pharmacological inhibition of HDAC6 markedly attenuates *DNHD1*-induced enhancements in cell proliferation, colony formation, migration and invasion, indicating that HDAC6 activity is required for *DNHD1*-mediated oncogenic effects in prostate cancer. (Scale bar, 100 µm). *P<0.05, **P<0.01, ***P<0.001. DNHD1, dynein heavy chain domain 1; HDAC6, histone deacetylase 6; Ac-α-tubulin, acetylated α-tubulin; SAHA, suberoylanilide hydroxamic acid (vorinostat); AR, androgen receptor; PSA, prostate-specific antigen; DMSO, dimethyl sulfoxide; Luc, luciferase; sh-Luc, short hairpin RNA targeting luciferase (control); shHDAC6, short hairpin RNA targeting histone deacetylase 6.

**Figure 6.**
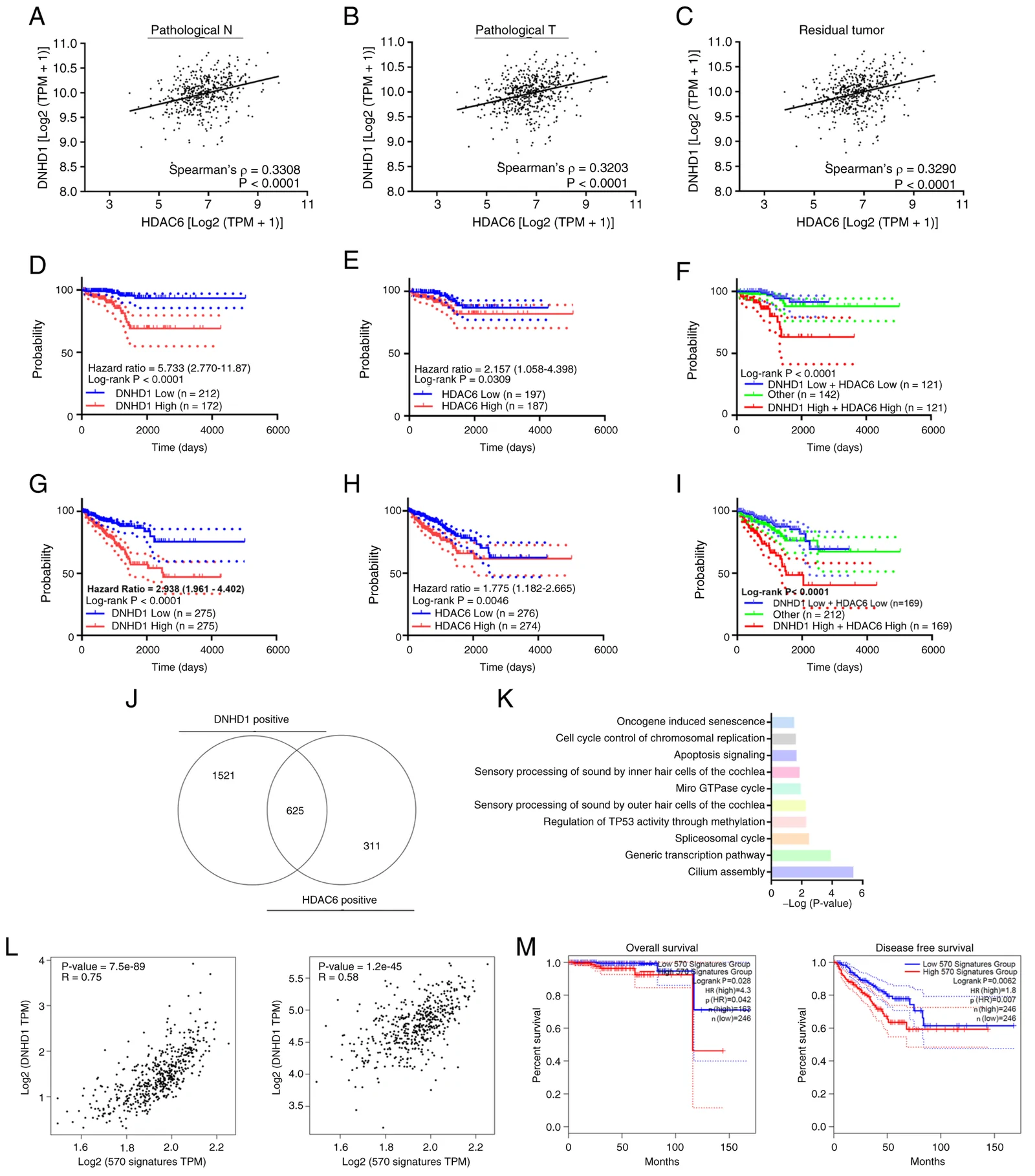
The DNHD1-HDAC6 axis is a transcriptionally coordinated program associated with prostate cancer progression and adverse clinical outcomes. Correlation analyses of the relationship between *DNHD1* and *HDAC6* expression across distinct clinicopathological contexts within the TCGA-PRAD cohort. Positive associations between *DNHD1* and *HDAC6* expression were observed in tumors stratified by pathological (A) N and (B) T stages and (C) residual tumor status, confirming the maintenance of the regulatory relationship between *DNHD1* and *HDAC6* during disease progression and posttreatment prostate cancer states. Kaplan-Meier analyses of DFI by (D) *DNHD1* and (E) *HDAC6* expression in patients from the TCGA-PRAD cohort. Patients were stratified into high- and low-expression groups on the basis of predefined cutoff criteria. (F) Integrated prognostic stratification of DFI based on combined *DNHD1* and *HDAC6* expression status. Coexpression analysis revealed a patient subgroup exhibiting the most unfavorable clinical outcomes. Kaplan-Meier analyses of PFI according to (G) *DNHD1* or (H) *HDAC6* expression levels in the TCGA-PRAD cohort. (I) Combined PFI analysis based on DNHD1-HDAC6 coexpression status demonstrating enhanced prognostic discrimination compared with single-gene stratification. (J) Venn-based integration of *DNHD1*- and *HDAC6*-associated transcriptional networks indicating a shared set of positively correlated genes that represent the common downstream regulatory program of the DNHD1-HDAC6 axis. (K) Functional enrichment analysis of the shared gene set associated with the DNHD1-HDAC6 axis. After exclusion of non-protein-coding transcripts, a refined 570-gene signature was established, with cilium assembly emerging as the most markedly enriched biological process. (L) Correlation analyses demonstrating significant positive associations between the DNHD1-HDAC6 downstream transcriptional signature and expression levels of *DNHD1* or *HDAC6* in PRAD specimens, confirming coordinated transcriptional coupling of the axis-associated gene program. (M) Kaplan-Meier analyses of OS and DFS according to the DNHD1-HDAC6 downstream transcriptional signature. Patients were stratified into high- and low-signature groups on the basis of predefined cutoff criteria, demonstrating the prognostic relevance of the shared DNHD1-HDAC6 gene program. DNHD1, dynein heavy chain domain 1; HDAC6, histone deacetylase 6; TCGA, The Cancer Genome Atlas; PRAD, prostate adenocarcinoma; TPM, transcripts per million; DFI, disease-free interval; PFI, progression-free interval; OS, overall survival; DFS, disease-free survival; HR, hazard ratio.

**Table I.** Multivariate Cox proportional hazards analysis for prostate cancer cohorts.

Clinical outcome/dataset	Covariate	b (Coeff)	SE	Wald	HR [(Exp(b))]	95% CI Lower	95% CI Upper	P-value	Significance
Disease-free	DNHD1	0.5901	0.1942	9.236	1.804	1.233	2.640	0.0024	^b^
interval	HDAC6	1.3566	0.6627	4.190	3.883	1.059	14.233	0.0407	^a^
(TCGA, n=384)	KLK3	0.1123	0.1670	0.452	1.119	0.807	1.552	0.5012	ns
Progression-	DNHD1	0.4325	0.1118	14.982	1.541	1.238	1.919	0.0001	^c^
free interval	HDAC6	0.8053	0.3444	5.468	2.238	1.139	4.395	0.0194	^a^
(TCGA, n=550)	KLK3	−0.0785	0.0636	1.526	0.925	0.816	1.047	0.2167	ns
Biochemically	DNHD1	0.0145	0.0068	4.527	1.015	1.001	1.028	0.0334	^a^
recurrent	HDAC6	0.0114	0.0043	7.198	1.012	1.003	1.020	0.0073	^b^
(GSE21032, n=113)	KLK3	−0.0001	0.0001	1.809	1.000	1.000	1.000	0.1787	ns

a P<0.05,

b P<0.01,

c P<0.001; ns, not significant. SE, standard error; HR, hazard ratio; CI, confidence interval; DNHD1, Dynein Heavy Chain Domain 1; HDAC6, Histone Deacetylase 6; KLK3, Kallikrein Related Peptidase 3; TCGA, The Cancer Genome Atlas; GSE, Gene Expression Omnibus Series; ns, not significant.

## Data Availability

The data generated in the present study may be found in the TCGA-PRAD project and GEO repository under accession numbers GSE21032, GSE35988, GSE48403 and GSE36133 or at the following URL: (persistent, direct URL to datasets). Additional data supporting the findings of the present study may be requested from the corresponding author.
