# Supplementary material for: Targeting the DNHD1-HDAC6 axis inactivates prostate cancer cell aggressiveness in vitro, suggesting potential therapeutic vulnerability
Source: Oncol Rep. 2026 Sep 30;56(5):198. doi: 10.3892/or.2026.9204 (PMC13659732; doi:10.3892/or.2026.9204)

Figure S1. E2F6 acts as a direct transcriptional regulator of *HDAC6* and mediates *DNHD1*-driven aggressive phenotypes in prostate cancer. (A) Upstream regulator analysis indicating E2F6 as a potential transcription factor involved in *DNHD1*-mediated *HDAC6* transactivation. (B) Correlation analyses of E2F6 expression with *DNHD1* and *HDAC6* in the TCGA-PRAD cohort (left and middle panels). Kaplan-Meier OS analysis comparing prostate cancer patients with high and low *E2F6* expression. Relative *HDAC6* mRNA expression determined through (C) qPCR and (D) relative *HDAC6* protein expression evaluated through immunoblotting after ectopic *E2F6* expression in LNCaP cells or *E2F6* knockdown in PC-3 cells. (E) Schematic representation of the *HDAC6* promoter region depicting the predicted E2F6-binding motif (5'-GAGAGGGAAGA-3'), located between positions -781 and -770 bp relative to the transcription start site. (F) Chromatin immunoprecipitation coupled with qPCR demonstrating the direct recruitment of E2F6 to the *HDAC6* promoter region in control and *E2F6*-overexpressing LNCaP cells, compared with the IgG control. (G) Immunoblotting of E2F6 and DNHD1 after reciprocal genetic manipulation (*DNHD1* overexpression or *E2F6* knockdown) in LNCaP cells. Quantification of (H) proliferation, (I) migratory potential and (J) invasive capacity in LNCaP cells subjected to *DNHD1* overexpression alone or in combination with *E2F6* knockdown. (K) Immunoblotting for E2F6 after *HDAC6* knockdown in LNCaP cells. (L) Colony formation, (M) Transwell migration and (N) Boyden chamber invasion assays in LNCaP cells cotreated with *E2F6* overexpression vector and *HDAC6*-targeting shRNA. (Scale bar, 100 μm). *P<0.05, **P<0.01, ***P<0.001. DNHD1, dynein heavy chain domain 1; HDAC6, histone deacetylase 6; E2F6, E2F transcription factor 6; TCGA, The Cancer Genome Atlas; PRAD, prostate adenocarcinoma; TPM, transcripts per million; OS, overall survival; HR, hazard ratio; mRNA, messenger RNA; qPCR, quantitative polymerase chain reaction; bp, base pairs; ChIP, chromatin immunoprecipitation; IgG, immunoglobulin G; Luc, luciferase; sh-Luc, short hairpin RNA targeting luciferase (control); sh-E2F6, short hairpin RNA targeting E2F transcription factor 6; sh-HDAC6, short hairpin RNA targeting histone deacetylase 6.

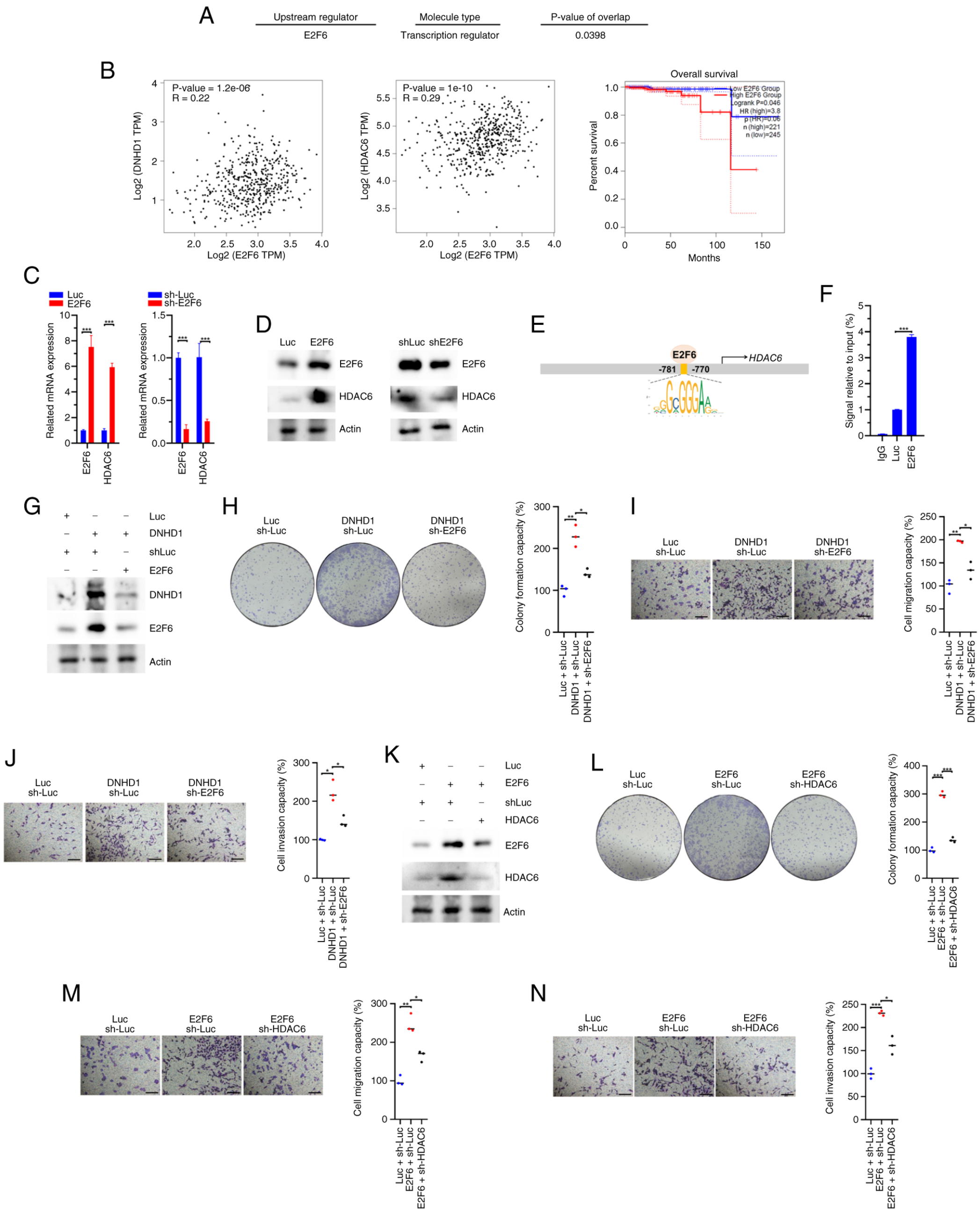

Supplement: Supplementary file 1 [file Supplementary_Data1.pdf]
